# 5-methyladenosine regulators play a crucial role in development of chronic hypersensitivity pneumonitis and idiopathic pulmonary fibrosis

**DOI:** 10.1038/s41598-023-32452-4

**Published:** 2023-04-12

**Authors:** Yiyi Zhou, Zhenli Hu, Qinying Sun, Yuchao Dong

**Affiliations:** Department of Respiratory and Critical Care Medicine, Changhai Hospital, Shanghai, China

**Keywords:** Computational biology and bioinformatics, Drug discovery, Immunology

## Abstract

5-methyladenosine (m5C) modification regulates gene expression and biological functions in oncologic areas. However, the effect of m5C modification in chronic hypersensitivity pneumonitis (CHP) and idiopathic pulmonary fibrosis (IPF) remains unknown. Expression data for 12 significant m5C regulators were obtained from the interstitial lung disease dataset. Five candidate m5C regulators, namely tet methylcytosine dioxygenase 2, NOP2/Sun RNA methyltransferase 5, Y-box binding protein 1, tRNA aspartic acid methyltransferase 1, and NOP2/Sun RNA methyltransferase 3 were screened using random forest and nomogram models to predict risks of pulmonary fibrosis. Next, we applied the consensus clustering method to stratify the samples with different m5C patterns into two groups (cluster A and B). Finally, we calculated immune cell infiltration scores via single-sample gene set enrichment analysis, then compared immune cell infiltration, related functions as well as the expression of programmed cell death 1 (PD-1, PDCD1) and programmed death protein ligand-1 (PD-L1, CD274) between the two clusters. Principal component analysis of m5C-related scores across the 288 samples revealed that cluster A had higher immune-related expression than B. Notably, T helper cell (Th) 2 type cytokines and Th1 signatures were more abundant in clusters A and B, respectively. Our results suggest that m5C is associated with and plays a crucial role in development of pulmonary fibrosis. These m5C patterns could be potential biomarkers for identification of CHP and IPF, and guide future development of immunotherapy or other new drugs strategies for pulmonary fibrosis.

## Introduction

CHP, one form of hypersensitivity pneumonitis (HP), is an inflammatory and/or fibrotic disease characterized by diverse histopathological and clinical features. Studies have shown that the disease, which is caused by immune-mediated responses to dominant or recessive inhaled antigens in susceptible individuals^1^ and causes serious morbidities and mortalities^2^, affects a wide range of humans, including older and younger individuals^3,4^. Notably, CHP patients present with symptoms similar to those observed in IPF counterparts, including chronic flu-like symptoms, dry cough, mid-inspiratory squeaks, dyspnea, chest tightness, and weight loss^1,5^. Approximately 11–65% of patients have radiographic findings of pulmonary fibrosis^6^. Pulmonary fibrosis can be easily confused with IPF^7,8^. Since subtypes of hypersensitivity pneumonitis, namely acute, subacute, and chronic, are still vaguely defined, there is need for clinicians to obtain patients’ clinical history, perform high-resolution computed tomography (HRCT), histopathology and multiple discipline diagnosis (MDD)^9,10^. The histopathological pattern of CHP includes a type called usual interstitial pneumonia (UIP), which exhibits a course similar to IPF. Studies have shown that this subtype not only causes clinically indistinguishable disease, but also delays recognition and avoidance of CHP stimulating antigen^11^. One study demonstrated that UIP/IPF is over-diagnosed and many of the cases so flagged were actually CHP with a UIP-like pattern^12^. The pathogenesis of IPF is repeated subclinical epithelial cell injury superimposed with accelerated epithelial aging, a phenomenon that results in abnormal repair of damaged alveoli and myofibroblast-induced interstitial fibrotic deposition^5^. To date, only a handful of studies have described similarities and differences among molecular mechanisms underlying occurrence and development of CHP and IPF. Understanding these mechanisms will greatly improve early screening of high-risk groups, identification of CHP and IPF, as well as development of targeted treatment therapies.

Modified nucleotides were discovered in large numbers of cellular RNAs as early as the 1960s^13^. Consequently, RNA modifications, including 5-methylcytosine (m5C), N1-methyladenosine (m1A), N6-methyladenosine (m6A), and uridylation (U-tail), have been shown to play a crucial role in modifying protein-coding messenger RNAs (mRNAs) and noncoding RNAs (ncRNAs)^14–17^. One such modification, termed m5C, has been shown to play a crucial role in RNA translation, transcription, and splicing^18,19^. RNA-modifying proteins (RMPs) that regulate m5C RNA methylation are called “writers”. These mainly include [members of the NOL1/NOP2/SUN domain (NSUNs) and DNA methyltransferase (DNMTs) families]^20,21^, “readers” [Aly/REF export factor (ALYREF) and Y-box binding protein 1 (YBX1)]^22^, and “erasers” [the ten-eleven translocator (TETs) family and AlkB homolog 1, histone H2A dioxygenase (ALKBH1)]^20^. Researchers have gradually focused their efforts on the importance of m5c modification in tumors, with evidence showing that high expression of NSUN2 is associated with poor prognosis in prostate cancer^23^. Another study demonstrated that NSUN2-deficient hepatocellular carcinoma cells were less in G1 and S phases, compared to G0 and G2 phases, indicating that inhibition of the NSUN2 gene significantly suppressed proliferation and division of HepG2 cells^24^. Moreover, DNMT1 was shown to be an epigenetic target for destruction and disruption metastatic and invasive phenotypes of TNBC cells^25^. To date, however, the expression pattern of m5C regulators in non-neoplastic diseases remains unknown.

In the present study, we hypothesized that m5C regulators regulate the mechanism underlying occurrence and development of CHP and/or IPF. To this end, we evaluated the functions of m5C regulators in diagnosis and classification of CHP and IPF based on the GSE150910 dataset. Our results reveal m5C regulator-mediated RNA methylation modification patterns and immune microenvironment infiltration characterization, and indicate that this genetic factor has potential as an immunotherapeutic agent.

## Methodology

### Data collection and processing

We used RNA-seq data from the NCBI Gene Expression Synthesis Database (NCBI–GEO, https://www.ncbi.nlm.nih.gov/geo), under accession number GSE150910. The tissues were isolated from surgical lung biopsy or lung transplants, including 103 unaffected controls, 103 and 82 patients IPF and CHP patients, respectively. We initially analyzed a total of 17 m5C regulators, including 11 writers (NOP2, TRDMT1, NSUN2-7, DNMT1, and DNMT3A-B), 2 readers (ALYREF, and YBX1) and 4 erasers (TET1-3 and ALKBH1). Apart from ALYREF, ALKBH1, NOP2, TET1 and TET3, we screened out 12 m5C regulators using differential expression analysis between patients (CHP, IPF) and their corresponding controls.

### Construction of random forest (RF) and nomogram models

RF is a combination of tree predictors that mitigates individual bias by combining and weighted regression or classification^26,27^. We systematically built a machine learning classifier, RF, to predict the occurrence of CHP and IPF, then used it to select candidate regulators from 12 m5C regulators. Furthermore, we examined the importance of the 22 m5C regulators and selected 5 with the highest scores for construction of a nomogram prognostic model for predicting prevalence of CHP and IPF.

### Consensus clustering and principal component analyses

To explore the relationship between m5C regulators with CHP and IPF, we stratified the GEO cohort into different subgroups according to the consensus level of m5C regulators. Next, we employed the “ConsensusClusterPlus” package in R to analyze and process the data, then visualized the results using consensus cumulative distribution function (CDF) plots, delta area plots and heatmaps. We also employed “ggplot2” package to generate a PCA plot and visualize the grouped data.

### Differential gene expression, and functional and pathway analyses

We employed the “limma” package in R to identify differentially expressed genes (DEGs) associated with m5C modifications, based on *P* < 0.05 and absolute value of log_2_FC (fold-change) > as cutoff. The identified DEGs were subjected to Gene Ontology (GO) functional enrichment, as well as Kyoto Encyclopedia of Genes and Genomes (KEGG) enrichment analysis using the “clusterProfiler” package and *P* value < 0.05as the threshold.

### Analysis of immune cell infiltration and function

We performed single sample gene set enrichment analysis (ssGSEA), which calculates an enrichment score to reveal the absolute enrichment level of a metagene set in a given dataset in each sample and gene set^28^, to count the abundance of immune cells in subgroups (cluster A and B). Immuno-correlation analysis was also performed based on ssGSEA and results visualized using heatmaps and boxplots. We then used the “estimate” package to perform ESTIMATE algorithm and generate a microenvironment score (immune score and stromal score), which was used to define the degree of immune cell infiltration and stromal cell infiltration, respectively.

### Identification of candidate small molecule drugs

To identify potential CHP/IPF therapeutic drugs, we divided DEGs associated with m5C typing into either down-regulated and up-regulated groups, then uploaded them to the Connectivity Map (CMap) database platform (https://clue.io/query), a platform for uncovering functional links between small molecule compounds, genes, and disease states^29,30^. We investigated the degree of similarity (which is represented by score and ranged from − 100 to 100) between the uploaded gene list and 2429 sets of small molecule processing expression profile data. Scores closer to 100 represents genes that were more similar to the small molecule treatment record. Conversely, a value closer to -100 denotes that this small molecule inhibits these genes.

### Statistical analysis

We employed the “biomaRt” package to filter the whole dataset, as well as delete duplicated and missing, then used the statistical procedure log2 (TPM) for data transformation. The correlation between writers and erasers was determined using linear regression, and the Wilcox-test applied to detect differences among several groups. Differences between CHP and IPF groups were compared using pairwise prop test. Model construction was achieved using “rms” and “rmda” packages in R. Model accuracy and validation were determined by plotting calibration, and clinical impact curves s, as well as decision curve analysis (DCA)”. All data was analysis procedures were performed using packages implemented R software versions 4.2.0 and R 4.2.1.

## Results

### Landscape of the m5C regulators in CHP and IPF

**Figure 1:**
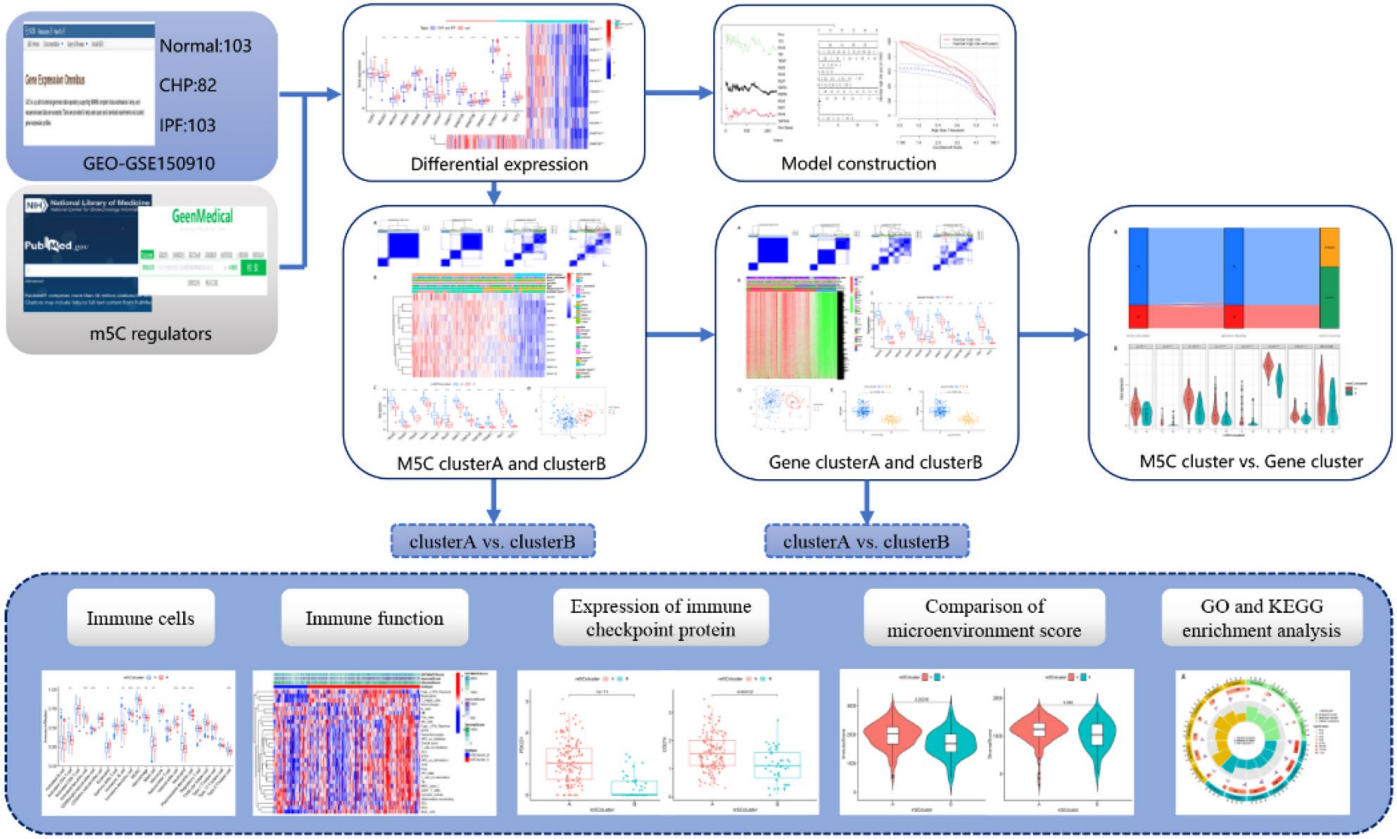
Flowchart of this study.

**Figure 2:**
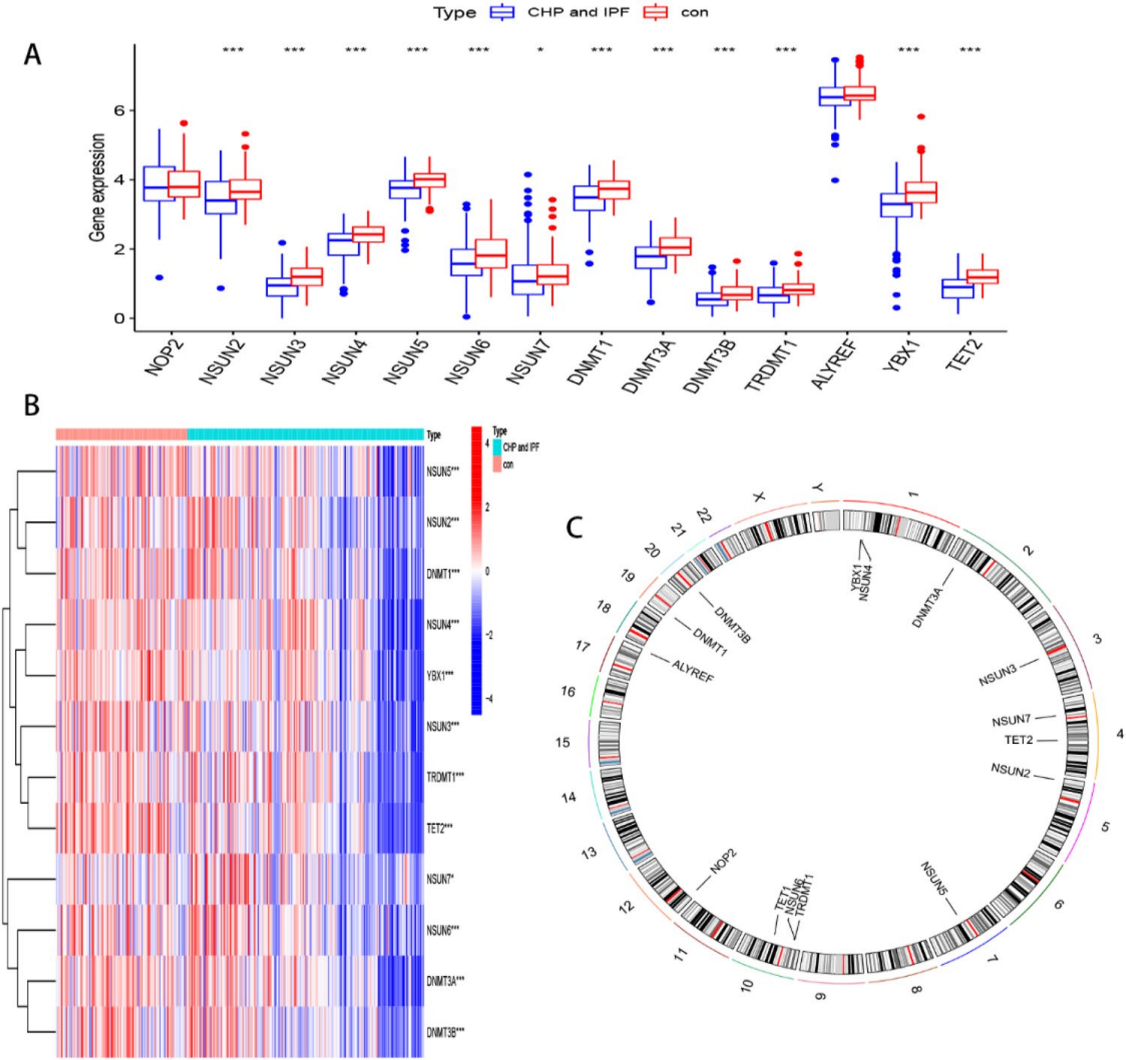
Landscape of the m5C regulators in CHP and IPF. (**A**) The boxplot of 14 m5C regulators expression in tests (CHP and IPF) and controls. (**B**) Expression of 12 significantly differentially expressed m5C regulators in tests and controls. *p < 0.05, **p < 0.01, and ***p < 0.001. (**C**) Chromosomal positions of those m5C regulators.

A summary of the study workflow is presented in Fig. 1, while profiles of differential expression among the 14 m5C regulators, between controls and tests (CHP and IPF), are illustrated in Fig. 2A. We screened out a total of 12 significant m5C regulators (DNMT1, DNMT3A-3B, NSUN2-7, TET2, TRDMT1, YBX1) (Fig. 2B), and found that all of them were significantly downregulated in experimental tissues, relative to corresponding controls. Profiles of chromosomal positions of the m5C regulators, as detected by the “RCircos” package, are shown in Fig. 2C.

### Erasers are correlated with writers

**Figure 3:**
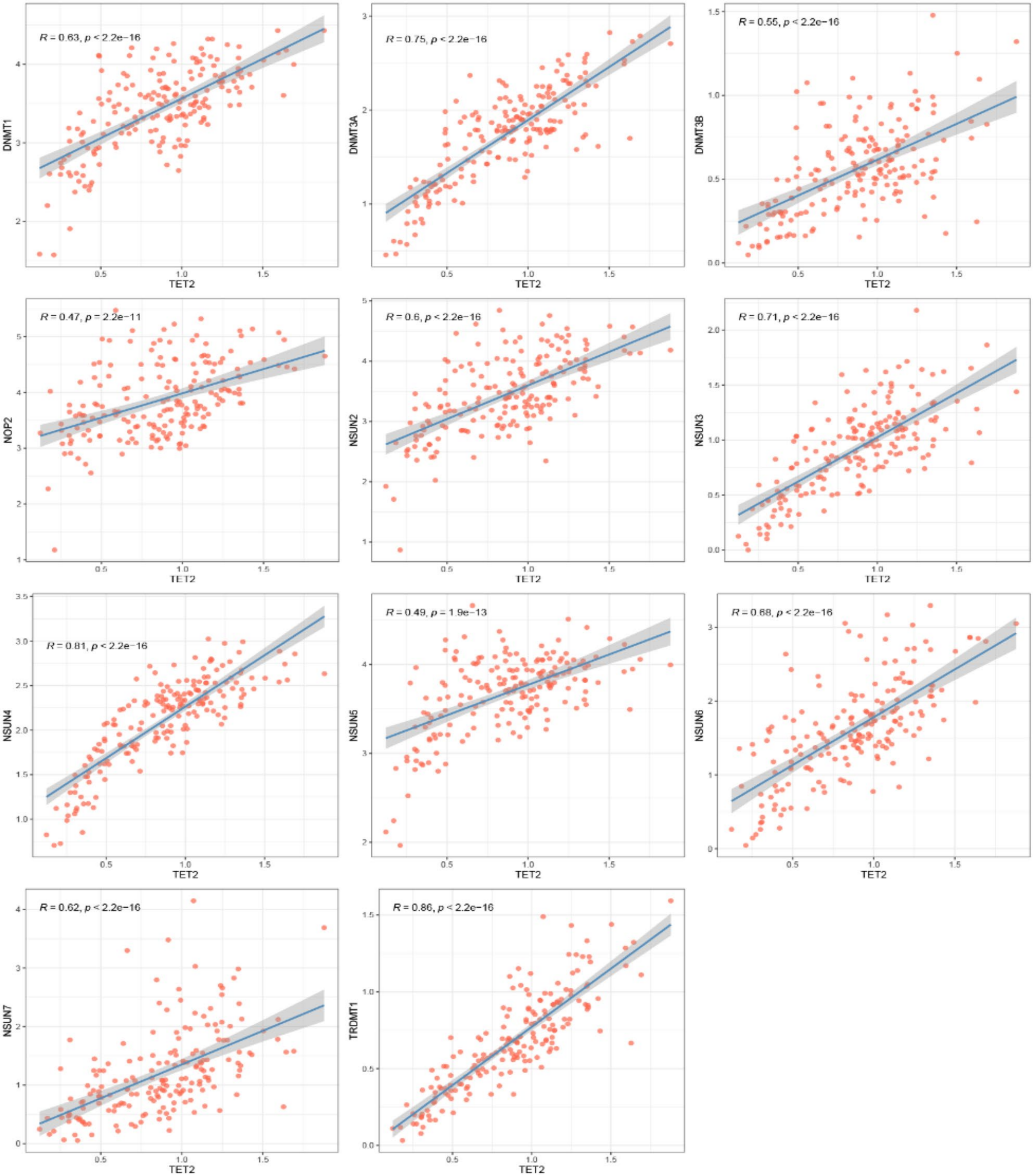
Correlation between m5C erasers and writers. Eraser gene: TET2. Writer genes: DNMT1, DNMT3A, DNMT3B, NOP2, NSUN2, NSUN3, NSUN4, NSUN5, NSUN6, NSUN7, TRDMT1. The threshold value: |R|> 0.4 and *P* < 0.001. R, correlation coefficient.

Genes cannot function in isolation, and there is evidence that m6A “writers”, “erasers”, and “readers” cooperate with each other in the context of cancer^31^. Therefore, the genetic expression correlations between m5C regulators were investigated. We used linear regression to explore the relationship among m5C “writers”, “readers” and “erasers”, and found a significant correlation between writers and erasers. Notably, the expression of 11 writers (DNMT1, DNMT3A-3B, NOP2, NSUN2-7, TRDMT1) was positively correlated with that of TET2 (Fig. 3). Correlation results are outlined in Table 1.

**Table 1:** The correlation results between erasers and writers.

Gene1	Gene2	cor	*p* value
TET2	NOP2	0.468168	1.82E−11
TET2	NSUN2	0.604137	8.71E−20
TET2	NSUN3	0.711152	8.19E−30
TET2	NSUN4	0.814944	3.18E−45
TET2	NSUN5	0.485332	2.53E−12
TET2	NSUN6	0.679229	2.24E−26
TET2	NSUN7	0.624686	2.06E−21
TET2	DNMT1	0.632135	4.94E−22
TET2	DNMT3A	0.752238	5.43E−35
TET2	DNMT3B	0.546126	8.97E−16
TET2	TRDMT1	0.861982	6.93E−56

### Construction of RF and nomogram models

**Figure 4:**
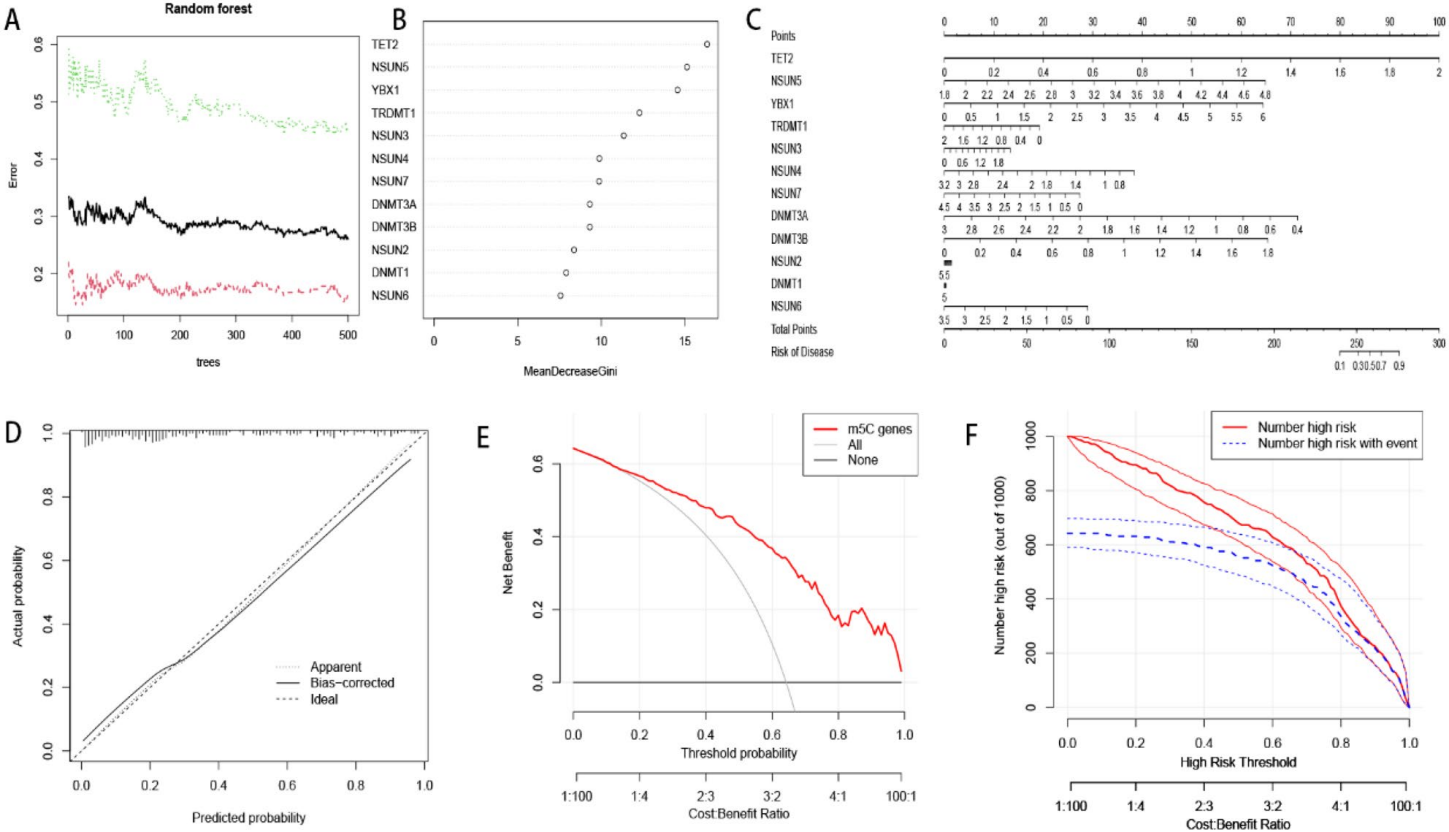
The RF and nomogram model construction. (**A**) The red line represented the error levels of treat groups, the green line represented control groups and the black line represented overall samples. (**B**) The importance of the m5C regulators were calculated based on the RF model. (**C**) The construction of nomogram model and gene score was used to predict prevalence. (**D**) The accuracy of nomogram model was assessed by calibration curve. (**E**) The decision curve might be benefit to the disease. (**F**) Clinical impact curve was applied for assessing clinical impact of the model.

We used the “randomForest” package construct a RF model comprising the top five m5C regulators (TET2, NSUN5, YBX1, TRDMT1 and NSUN3) to predict occurrence of CHP and IPF. Profiles of the M5C regulators, visualized after ranking these genes according to their importance, are presented in Figs. 4A,B. The constructed nomogram is presented in Fig. 4C. Notably, there is a short distance between the dashed and solid lines in calibration curve (Fig. 4D), as well as the clinical impact curve (Fig. 4F), indicating that the nomogram was highly accurate. The following red line in DCA curve staying above the grey and black lines reflected benefit to CHP and IPF (Fig. 4E).

### Identification of the two m5C clusters

**Figure 5:**
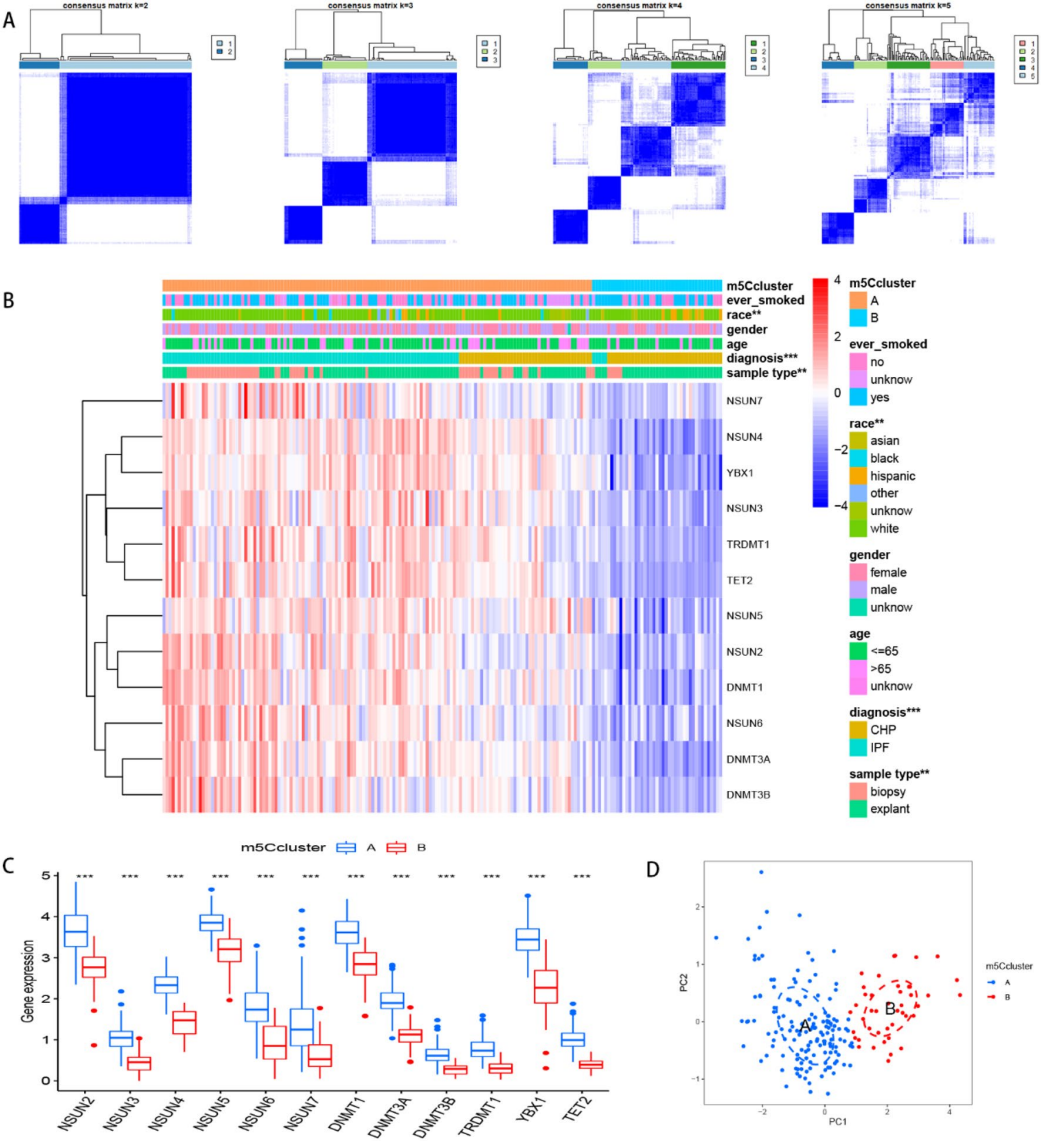
Consensus clustering of m5C regulators. (**A**) Consensus matrices of the 12 m5C regulators (k = 2–5). (**B**) Expression of 12 significant m5C regulators in the heatmap. (**C**) Expression of 12 significant m5C regulators in the boxplot. (**D**) PCA showed the striking difference in modification cluster A (the blue pattern) and cluster B (the red pattern). *p < 0.05, **p < 0.01, and ***p < 0.001.

Next, we employed consensus clustering analysis to distinguish among m5C patterns. As illustrated in Fig. 5A, we divided the tests (IPF and CHP) into 2 clusters (m5C cluster A and cluster B) according to the discrepancy analysis result file gained from Fig. 2B. Discrepancies between analysis result file and clustering results are presented in Supplementary Tables S1, S2, respectively. Notably, all the 12 m5C regulators (DNMT1, DNMT3A-3B, NSUN2-7, TET2, TRDMT1 and YBX1) had significantly higher expression levels in cluster A than B (Figs. 5B,C). We further visualized the correlation between clinical characteristics and m5C clusters using a heatmap. To this end, it was evident that patients in cluster A tended to be more diagnosed with IPF, and also had more lung biopsies and diverse races compared to their counterparts in cluster B. These results were verified using PCA (Fig. 5D).

**Figure 6:**
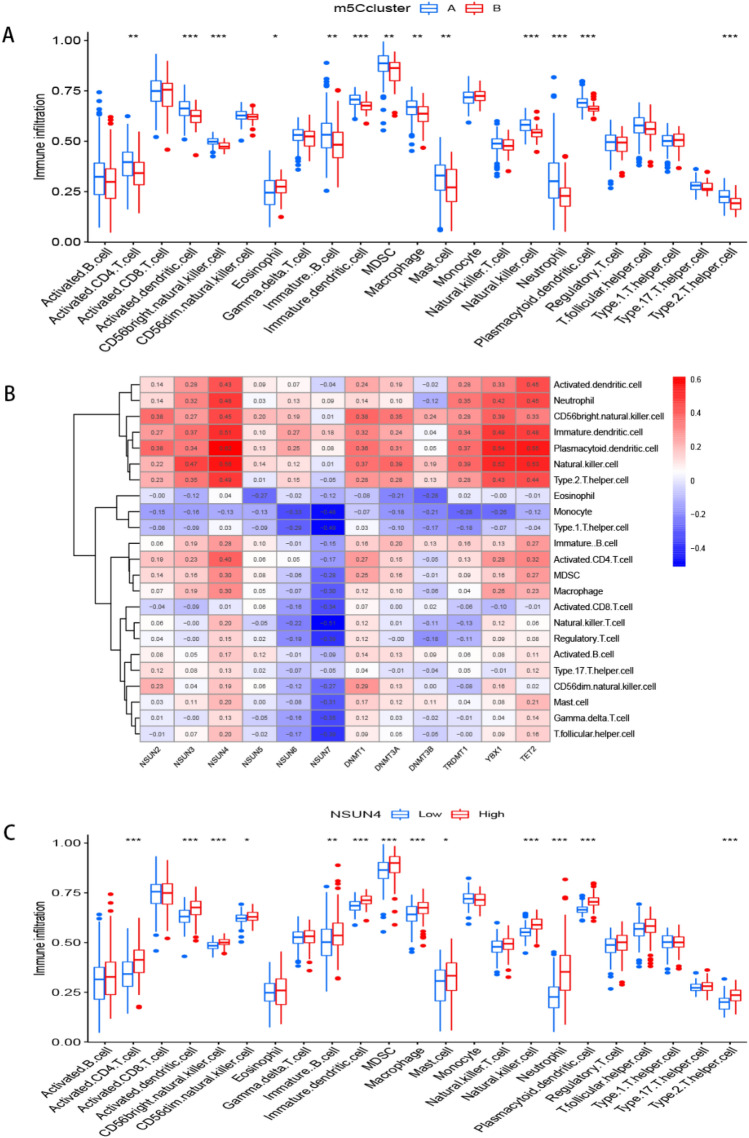
Single sample gene set enrichment analysis of immune cells infiltration. (**A**) The relationship between immune cells infiltration with two m5C patterns. (**B**) The heatmap of the 12 significant m5C regulators and infiltrating immune cells. (**C**) Immune cell infiltration between high NSUN4 expression pattern and low NSUN4 expression pattern. *p < 0.05, **p < 0.01, and ***p < 0.001.

Next, we calculated the abundance of immune cells in clusters A and B and found that cluster A was significantly associated with Th2 cells (*p* < 0.001) and mast cells (MCs) (*p* < 0.01) than B. On the other hand, cluster B was more closely associated with eosinophils (*p* < 0.05) (Fig. 6A). It can be seen from Fig. 6B that all the 12 m5C regulators (DNMT1, DNMT3A-3B, NSUN2-7, TET2, TRDMT1 and YBX1) had negative association with monocyte and positive association with CD56^bright^ natural killer cell, immature dendritic cell, plasmacytoid dendritic cell and natural killer cell. We also observed that NSUN4 was positively correlated with those immune cells (Fig. 6B), which inspired us to explore the relationship between NSUN4 expression and immune cell infiltration. Results showed that patients with high NSUN4 expression also had elevated immune cell infiltration (Fig. 6C).

**Figure 7:**
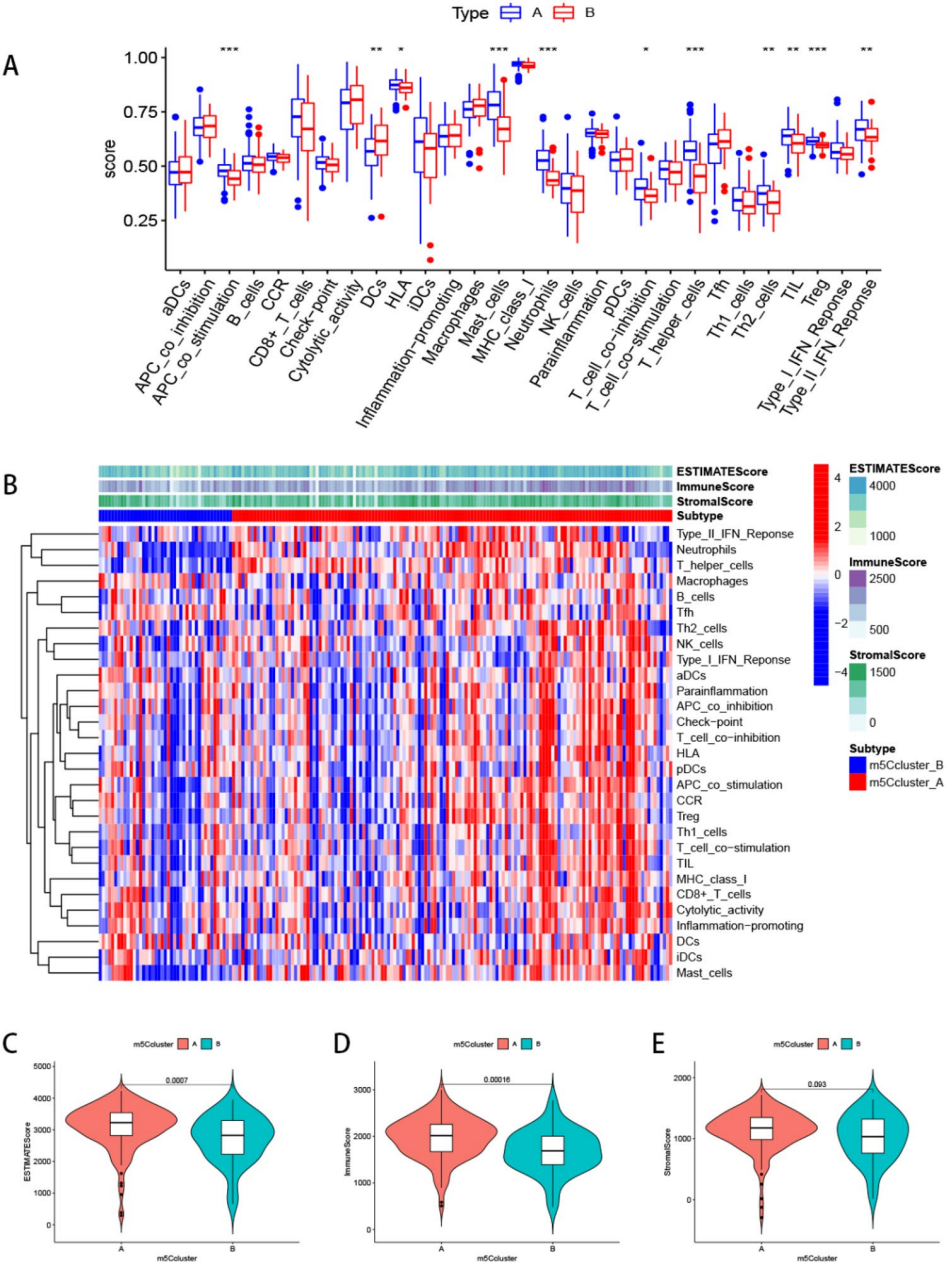
Single sample gene set enrichment analysis of the two m5Cclusters. (**A**) The boxplot of immune function in the two m5C patterns. *p < 0.05, **p < 0.01, and ***p < 0.001. (**B**) The heatmap of immune function in the two m5C patterns. Other characteristics including estimate score, immune score, and stromal score. Differential estimate score (**C**), immune score (**D**), and stromal score (**E**) between m5Ccluster A and B.

**Figure 8:**
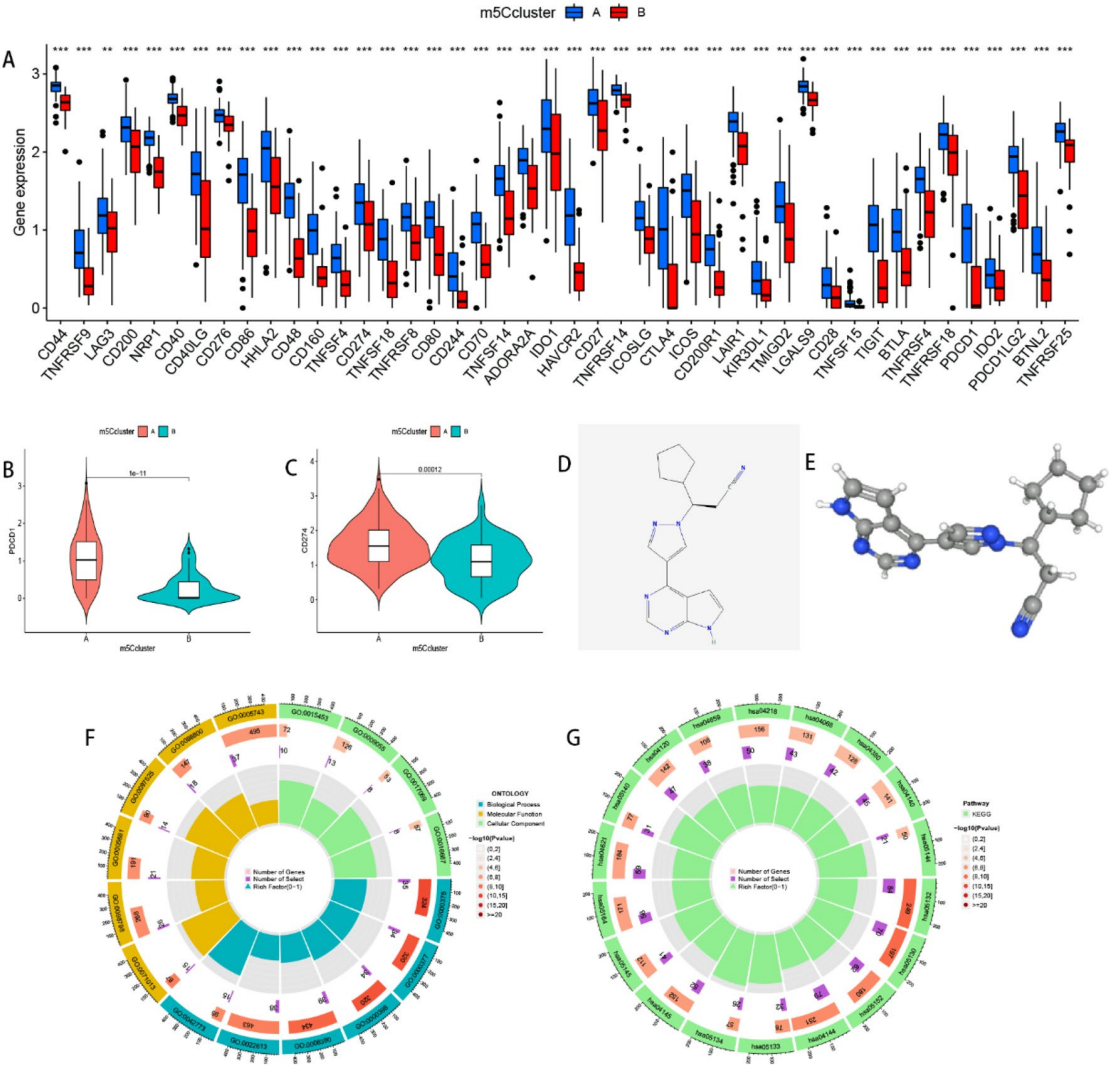
Analyses of immune checkpoints, small molecule drug therapy, GO, as well as KEGG Pathway in two m5Cclusters. (**A**) The boxplot of immune checkpoints in the two m5C patterns. *p < 0.05, **p < 0.01, and ***p < 0.001. (**B**) The expression of PDCD1 (PD-1) in the two m5C patterns. (**C**) The expression of CD274 (PD-L1) in the two m5C patterns. Two-dimensional molecular structure (**D**) and three-dimensional molecular structure (**E**) of ruxolitinib. The GO (**F**) and KEGG (**G**) enrichment analysis for the m5C-related differentially expressed genes (DEGs).

Next, we employed the “GSVA” and “GSEABase” packages to determine immune-related functions in the two clusters, and presented the results using boxplots and heatmaps. Results showed that the 11 types of immune function had different profiles across the subtypes (Figs. 7A,B). Apart from dendritic cells (DCs), it was evident that cluster A had markedly higher immune activity than cluster B. The m5C cluster A got higher immune score (*p* = 0.00016) and stromal score (*p* = 0.093) than cluster B (Figs. 7C–E). Considering the relevance of immune checkpoints to immunotherapy, we studied the immune checkpoint expression and found that all the 47 immune checkpoint genes (ICGs) were significantly overexpressed in cluster A relative to cluster B (Fig. 8A). Previous studies on pulmonary fibrosis have shown that PD-1/PD-L1 promotes the development of pulmonary fibrosis through different pathways^32–35^. Therefore, generated violin plots to visualize PD-1/PD-L1 and found that it was significantly upregulated in cluster A (*p* < 0.001) (Figs. 8B,C).

GO and KEGG analyses revealed that a total of 3346 DEGs between m5C cluster A and cluster B have relevance in small molecule drug screening (Supplementary Table S3). Next, we selected the top 11 genes, based on adj. *P* values as well as removal of invalid genes and valid but not used genes in query, were selected from each of the upregulated DGEs and downregulated DEGs (|lgFC|> 2.4 and adj. *P* < 0.001). Results of the top 11 genes are shown in Supplementary Tables S4, S5, while the structure of ruxolitinib is presented in Fig. 8D,E. The top 10 strongest inhibitory small molecule drugs are outlined in Table 2.

**Table 2:** Top 10 strongest inhibitory small molecule drugs.

Name	ID	Score	Description
ruxolitinib	BRD-K53972329	− 98.91	JAK inhibitor
orantinib	BRD-K91696562	− 98.77	FGFR inhibitor
UNC-0321	BRD-K74236984	− 98.72	Histone lysine methyltransferase inhibitor
AR-A014418	BRD-K67860401	− 97.64	Glycogen synthase kinase inhibitor
doxercalciferol	BRD-K14550461	− 97.11	Vitamin D receptor agonist
PT-630	BRD-A73680854	− 96.76	Dipeptidyl peptidase inhibitor
BRL-50481	BRD-K84266862	− 96.71	Phosphodiesterase inhibitor
rolipram	BRD-A34255068	− 96.61	Phosphodiesterase inhibitor
actarit	BRD-K33483813	− 95.39	Interleukin receptor agonist
forskolin	BRD-A70449690	− 95.09	Adenylyl cyclase activator

GO analysis revealed that the DEGs were mainly enriched in RNA splicing (GO:0008380), RNA splicing via transesterification reactions (GO:0000375), mitochondrial inner membrane (GO:0005743), and electron transfer activity (GO:0009055) (Fig. 8F). On the other hand, KEGG pathway analysis showed that the DEGs were mainly involved in NOD—like receptor signaling (hsa04621) and FoxO signaling (hsa04068) pathways (Fig. 8G).

### Identification of the two gene clusters

**Figure 9:**
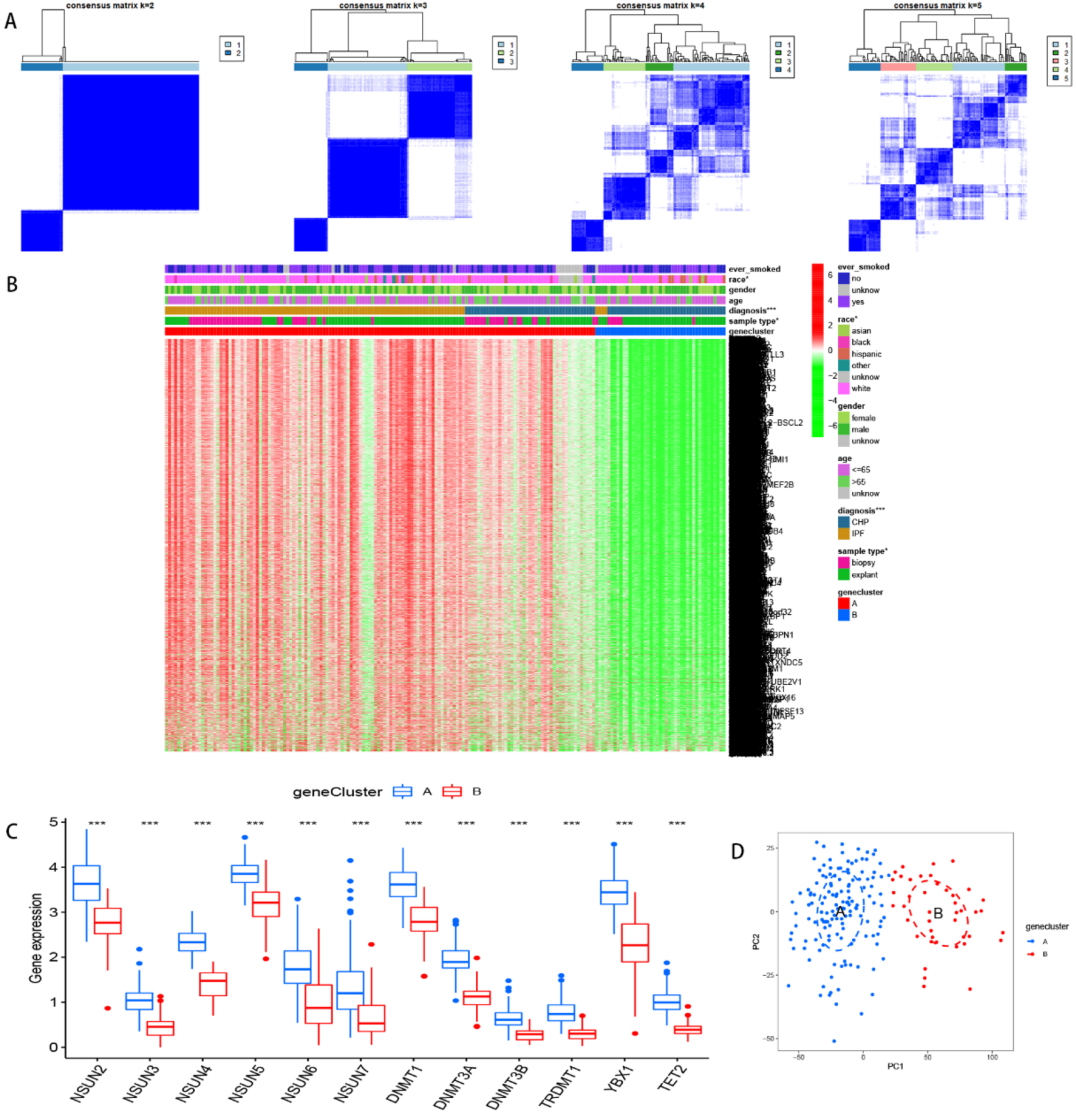
Consensus clustering of the 3346 m5C-related DEGs. (**A**) Consensus matrices of the DGEs (k = 2–5). (**B**) Expression of the DEGs in gene cluster A and cluster B. (**C**) Expression of the 12 m5C regulators in gene cluster A and cluster B. (**D**) Principal component analysis for the expression profiles of gene subtypes, also showing a remarkable difference between different modification patterns. *p < 0.05, **p < 0.01, and ***p < 0.001.

**Figure 10:**
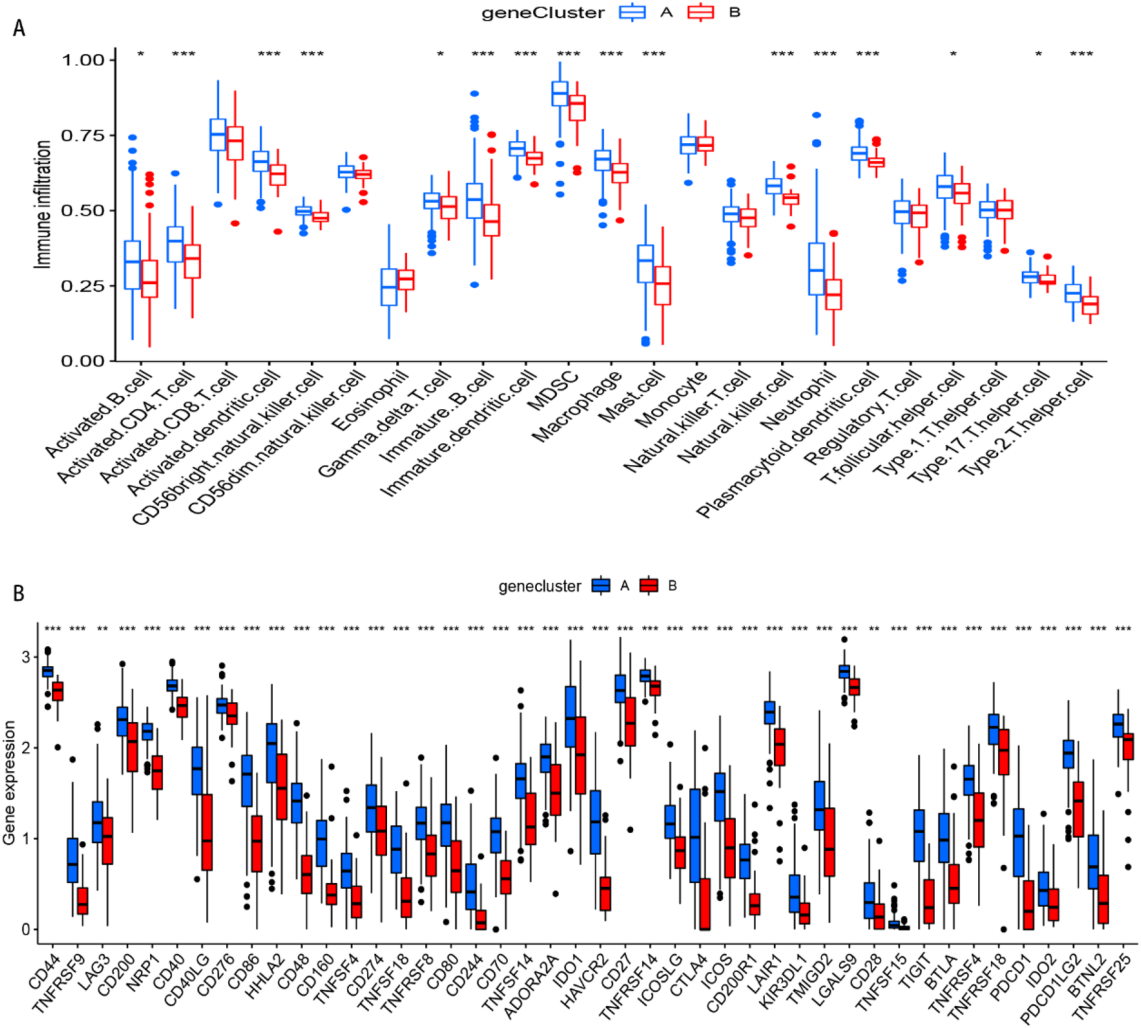
Analyses of immune cells infiltration and immune checkpoints in two m5Cclusters. (**A**) The relationship between immune cells infiltration with two m5C patterns. (**B**) The boxplot of immune checkpoints in the two m5C patterns. *p < 0.05, **p < 0.01, and ***p < 0.001.

**Figure 11:**
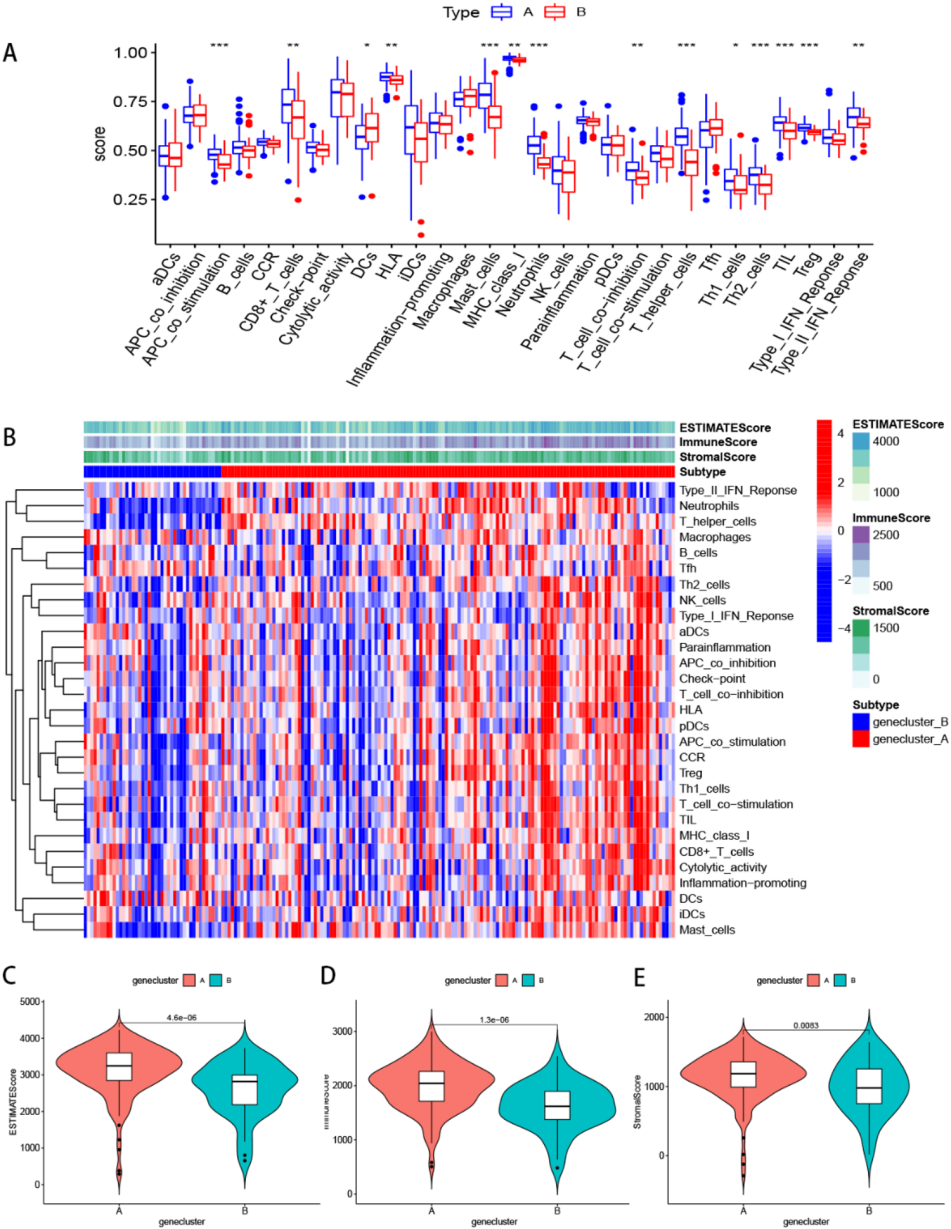
Single sample gene set enrichment analysis of the two gene clusters. (**A**) The boxplot of immune function in the two gene patterns. *p < 0.05, **p < 0.01, and ***p < 0.001. (**B**) The heatmap of immune function in the two gene patterns. Other characteristics including estimate score, immune score, and stromal score. Differential estimate score (**C**), immune score (**D**), and stromal score (**E**) between m5Ccluster A and B.

Next, we used consensus cluster analysis to distinguish among DEGs patterns, and divided the DEG patterns into 2 clusters, 3 clusters, 4 clusters or 5 clusters, and identified 2 clusters (gene cluster A and cluster B) (Fig. 9A). Summarily, DEGs and 12 m5C regulators (DNMT1, DNMT3A-3B, NSUN2-7, TET2, TRDMT1 and YBX1) were significantly upregulated in cluster A than B (Figs. 9B,C). A similar pattern was observed with regards to m5C patterns, where patients in cluster A tended to be diagnosed more with IPF, exhibit more lung biopsies and diverse races compared to their counterparts in B. These findings were confirmed by PCA plots (Fig. 9D). In IPFaddition, gene cluster A was associated with immune cell infiltration, and cluster A was significantly associated with Th2 cells and MCs than B (*p* < 0.001) (Fig. 10A). We also studied expression of immune checkpoints and found a significantly higher expression of all the 47 ICGs in cluster A relative to B (Fig. 10B). Differences across the subtypes are presented by boxplots and heatmaps in Figs. 11A,B. Apart from dendritic cells (DCs), it was evident that cluster A had higher immune activity, immune scores (*p* = 1.3e−06) and stromal scores (*p* = 0.0083) than cluster B (Figs. 11C–E).

### Comparison in m5C and gene patterns

**Figure 12:**
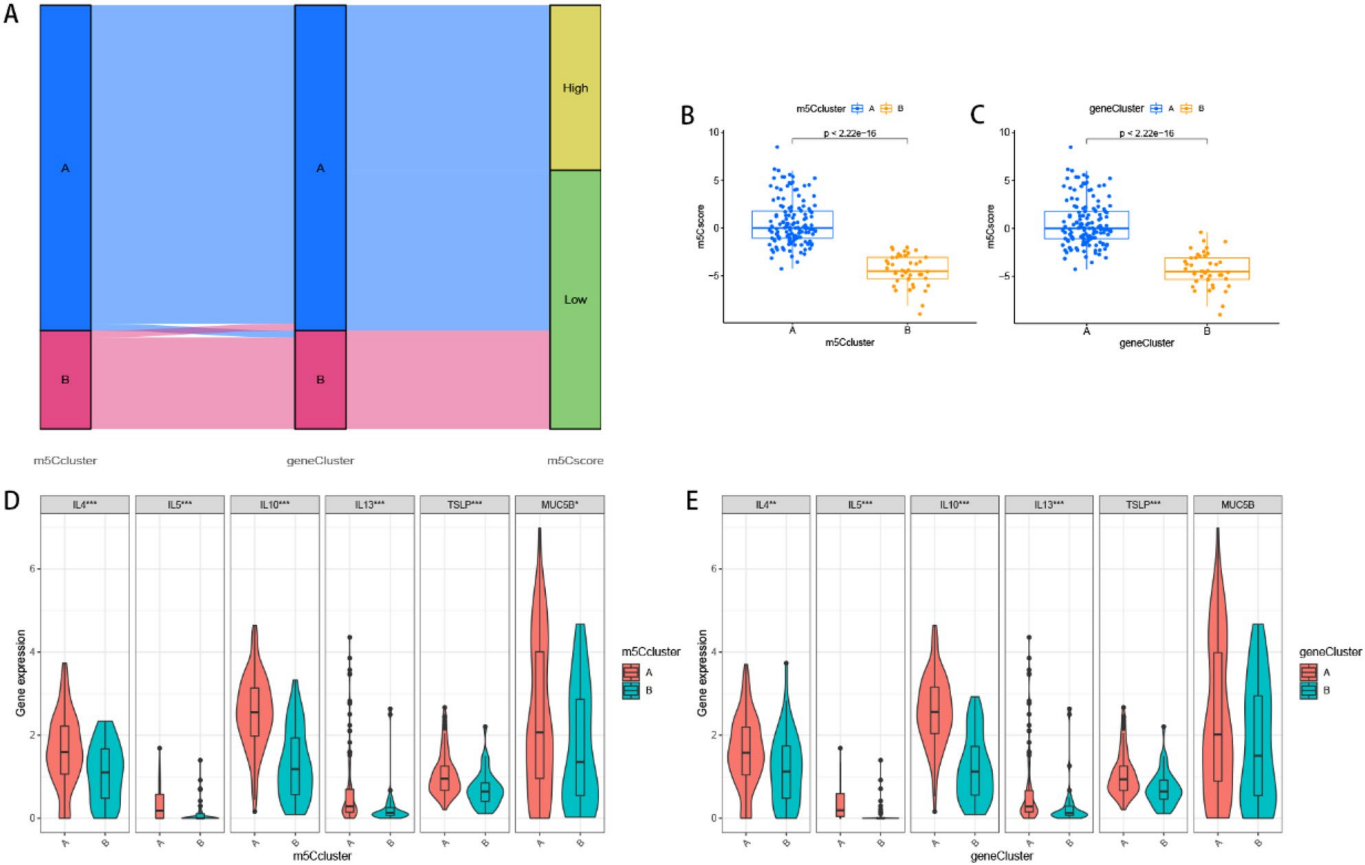
Comparison of m5C patterns and gene patterns. (**A**) Sankey diagram of the relationship between two m5C patterns, two gene patterns, and m5C scores. Differences in m5C score based on PCA algorithm between the two m5C patterns (**B**) or the two gene patterns (**C**). Differential expression levels of IL-4, IL-5, IL-10, IL-13, TSLP and MUC5B between m5C cluster A and cluster B (**D**) or gene cluster A and cluster B (**E**). *p < 0.05, **p < 0.01, and ***p < 0.001.

We generated a PCA plot to depict m5C-related scores across samples. Summarily, m5C and gene typing were analogous, with the sankey diagram distinctly indicating that the clusters were associated with m5C-related scores (Fig. 12A). Notably, cluster A exhibited significantly higher scores than cluster B (Figs. 12B,C). Previous studies have suggested that high mucin 5B (MUC5B) expression is associated with honeycombing and fibrosis in both CHP and IPF subjects^11,36,37^, combing our earlier results suggested a strong link between m5C/gene cluster A and Th2 cells. Consequently, we analyzed the relation between m5C clusters and Th2 cytokines [Interleukin (IL) -4, IL-5, IL-10, IL-13, and thymic stromal lymphopoietin (TSLP)], as well as MUC5B, and found a significant positive association (Figs. 12D,E). Notably, cluster A recorded higher expression levels than B, indicating that the former was highly linked to honeycombing and fibrosis, as well as lung fibrosis characterized by the Th2 immune response.

## Discussion

In our previous study, we demonstrated that two distinct m6A patterns were associated with fibrosis and might distinguish CHP and IPF^38^. Inspired by these findings, we analyzed expression patterns of the major m5C regulators altered in GSE150910 and found that they played a key role in occurrence and development of CHP and IPF. Firstly, we explored the correlation between “writers”, “erasers” and “readers” and found a significant relationship between various writers and one eraser. Next, we used five candidate m5C regulators to construct a nomogram model for predicting the prevalence of CHP or IPF patients. Results indicated that this model was effective in diagnosis and treatment of these patients. Finally, we studied various methylation modification patterns to explore two distinct m5C patterns and hypothesized the two patterns may associate with lung fibrosis and distinguish patients with CHP or IPF.

Analysis of clinical characteristics, immune microenvironment, and potential drug therapies were performed. Clinical characteristics in the two m5C patterns revealed more IPF patients in m5C cluster A than cluster B, implying that these two patterns might distinguish IPF from CHP. Previous studies have shown that HP is mediated by Th1 cytokine immunity^39^ and Th2 is related to formation of pulmonary fibrosis^40–43^. MCs cooperate with fibroblasts to promote pulmonary fibrosis^44^, and accumulation of MCs has been found in HP^45^, IPF^44,46^, sarcoidosis^47^ and silicosis^48^. Moreover, Cha SI et al. found the MCs in IPF is more than CHP^46^. In our study, more Th2 cells and MCs aggregation in cluster A further implied that CHP and IPF could be distinguished by the two patterns, and the mechanism of fibrosis might be fundamentally different between CHP and IPF. Accumulation of eosinophils in cluster B was consisted with the deduction^49,50^. Next, we compared expression levels of Th2-related cytokines between the clusters and found a higher expression in cluster A than B. Besides, MUC5B promoter variant rs35705950 is a high-risk factor for IPF^11^, the cluster A exhibited higher MUC5B expression than B. These results suggested that cluster A was related to IPF. We also found higher immune and stromal scores in m5C cluster A than B, implying that immune element plays an underlying impact on cluster A. PD-1/PD-L1 was significantly upregulated in cluster A than B, indicating that patients in the former cluster were more likely to promote fibrosis and have better immune efficacy than their cluster B counterparts.

GO analysis revealed that the identified DEGs were mainly enriched in NOD—like receptor and FoxO signaling pathways, which have been shown to play important roles in inflammatory response^51,52^, indicating that their immune components involved in the pathological processes and immunoregulatory mechanisms of CHP and IPF. We found two distinct DEGs-related patterns, with further analysis of clinical characteristics and immune microenvironment revealing patterns that were consistent with those observed in m5C. In general, the two m5C patterns have potential to distinguish patients with CHP from IPF.

The present study had some limitations. Firstly, the GSE150910 used herein lacked data on pathological classification and imaging features for patients with CHP or IPF. Although the probability of misdiagnosis in these samples was low, it cannot be ruled out. Secondly, although we searched and downloaded all interstitial pneumonia datasets from the GEO database, no other dataset contained both IPF and CHP, thus we had no validation dataset. In future, we expect to use NGS of clinical specimens coupled with experimental studies based on pulmonary fibrosis mouse models to validate these findings.

## Conclusion

In summary, our findings revealed the role of m5C methylation regulators in development of pulmonary fibrosis. These genetic factors have potential in distinguishing CHP and IPF patients, thus are expected to be used in developing new immunotherapy strategies for these patients.

## Supplementary Information

Supplementary Information 1.

Supplementary Information 2.

Supplementary Information 3.

Supplementary Information 4.

Supplementary Information 5.

## Data Availability

The dataset is available in online repositories (NCBI–GEO, https://www.ncbi.nlm.nih.gov/geo), under accession number GSE150910. All data to support our conclusions are included in this article.
